# Multicystic Benign Cystic Mesothelioma Presenting as a Pelvic Mass

**DOI:** 10.1155/2014/852583

**Published:** 2014-03-12

**Authors:** Mazdak Momeni, Elena Pereira, Gennadiy Grigoryan, Konstantin Zakashansky

**Affiliations:** Division of Gynecologic Oncology, Department of Obstetrics, Gynecology and Reproductive Medicine, Icahn School of Medicine at Mount Sinai Medical Center, 1176 Fifth Avenue, P.O. Box 1173, New York, NY 10029-6574, USA

## Abstract

*Background.* Benign cystic mesothelioma (BCM) is a rare tumor that arises from the abdominal peritoneum with a predilection to the pelvic peritoneum. For this reason, it can often mimic gynecologic malignancies. *Case.* A 47-year-old perimenopausal female presented reporting several weeks of abdominal distention associated with abdominal tenderness and constipation. Computed tomography revealed a 24 cm multiloculated pelvic mass, and tumor markers were notable for an elevated CA-125. The patient was taken to the operating room for an exploratory laparotomy, total abdominal hysterectomy, bilateral salpingoophorectomy, and removal of pelvic mass. Final pathologic evaluation revealed a benign cystic mesothelioma. *Conclusion.* Classically these tumors present as large multicystic masses with thin-walled septations and on preoperative evaluation BCM can mimic many different disease entities including ovarian malignancies and cystic lymphangioma. Often diagnosis can only be made at time of surgery.

## 1. Introduction

Differential diagnosis of a pelvic mass in a reproductive age female can be difficult and often requires surgical exploration and pathologic diagnosis. Benign cystic mesothelioma is a rare intra-abdominal tumor that can present as a large multicystic mass arising from the pelvis. To date, approximately 140 cases have been described in the literature, most occurring in reproductive aged women. The pathogenesis of this disease remains unclear, although many agree that it is likely the result of a chronic inflammatory process as in the case of endometriosis. BCM is generally considered a benign process; however given the high rate of recurrence and possible malignant transformation, close follow-up is important.

## 2. Case Report

Written informed consent for publication was first obtained from the patient as outlined by the Institutional Review Board. A 47-year-old perimenopausal female presented to her gynecologist's office complaining of worsening abdominal distention, abdominal tenderness, and constipation for over 3 weeks. Past medical and surgical history was significant for hypothyroidism, latent tuberculosis, and cesarean section with bilateral tubal ligation. She had no smoking history and family medical history was noncontributory. On physical exam, vital signs were within normal limits and the abdomen was distended and diffusely tender. Pelvic exam was significant for a 30 cm adnexal mass. The uterus was small and difficult to palpate secondary to the large adnexal mass. The remainder of the physical exam and review of systems were otherwise unremarkable.

**Figure 1:**
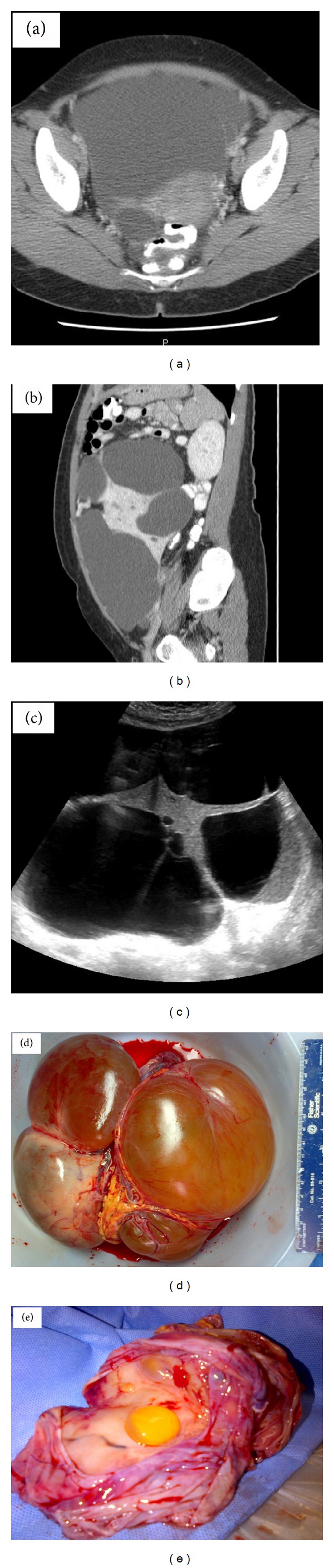
(a) CT image: axial view. (b) CT image: sagittal view. (c) Ultrasound image: transvaginal. (d), (e) Photo: gross pathology.

Transvaginal and transabdominal ultrasound with color Doppler was performed. Findings were notable for a large pelvic mass likely originating in the left ovary and extending to the level of the first intercostal space. The mass was described as a multiloculated complex with multiple thick septations measuring 24 × 18 cm (Figure 1). Moderate free fluid was also noted in the pelvis. The uterus was anteverted and normal in appearance with a thin endometrial lining. The right ovary measured 3.9 × 3.3 × 3.5 cm with a 3 cm simple cyst. Computed tomography (CT) scan revealed a 28.3 × 24.4 × 16.3 cm predominantly cystic mass extending from the pelvis to the upper abdomen with displacement of the bowel superiorly (Figure 1). Additional findings described were similar to those noted on pelvic ultrasound. Tumor markers were sent and CA125 was significantly elevated, with a value of 95.

The patient underwent an exploratory laparotomy, pelvic mass excision, partial omentectomy, adhesiolysis, total abdominal hysterectomy, and bilateral salpingoophorectomy. There was high suspicion for malignancy and a gynecologic oncologist was present at time of surgery. Surgical findings included a normal uterus, fallopian tubes, and ovaries. The large 26–28 cm multiloculated peritoneal cyst was noted to be originating from the left cornual region of the uterine fundus from a wide pedicle (Figure 1). There were significant adhesions involving the cyst, ureters, omentum, and pelvic sidewall. Three liters of cystic fluid was evacuated and sent for cytology. Gross pathologic evaluation of the specimen described the specimen as consisting of solid and multicystic areas. Cytology was significant for reactive benign mesothelial cells. After histologic evaluation a diagnosis of multilocular peritoneal inclusion cyst (benign cystic mesothelioma) was given.

Her postoperative course was unremarkable and the patient was discharged home on the third hospital day. She has had several follow-up visits and at 1 year after surgery she has no new complaints and no evidence of recurrence.

## 3. Discussion

Benign cystic mesothelioma (BCM) is a rare tumor that arises from the abdominal peritoneum. Classically these tumors present as large multicystic masses with thin-walled septations and predilection to the pelvic peritoneum [1]. Typical symptoms include abdominal distention, abdominal tenderness, ascites, nausea, and constipation. To date, approximately 140 cases have been described in the literature, most occurring in reproductive aged women [1–4]. Given the unclear origin of this disease it appears in the literature under various names including: multilocular peritoneal inclusion cysts, multicystic peritoneal mesothelioma, and postoperative peritoneal cysts [5].

On preoperative evaluation BCM can mimic many different disease entities including ovarian malignancies and cystic lymphangioma. Often diagnosis can only be made at time of surgery. On ultrasound BCM typically appears as a multicystic, vascular mass without calcifications. CT scan and magnetic resonance imaging (MRI) scan typically reveal multilocular cystic lesions, with thin-walled septations and fluid densities [1, 4]. Fine-needle biopsy can be considered but is often nonspecific and demonstrate only reactive mesothelial cells [1]. Additionally, it is generally not advisable to aspirate pelvic masses that are suspicious for ovarian malignancy, as rupture of the cyst wall will worsen overall prognosis.

Although reports of BCM first appeared in the literature in 1979 the pathogenesis of this disease remains unclear [6]. In female patients, there appears to be an association with BCM and endometriosis, pelvic inflammatory disease, and a history of abdominal surgeries [4, 7]. In fact, when considering endometriosis as an inciting factor, several authors have demonstrated the presence of estrogen and progesterone receptors in normal peritoneum and have proposed the use of gonadotropin-releasing hormone agonists as therapy [1, 4, 8–10]. Several additional hypotheses have been proposed with regard to pathogenesis. It is generally accepted that the underlying cause of disease is chronic peritoneal inflammation, which causes the proliferation and migration of peripheral mesothelial cells along with metaplasia of underlying connective tissues. The use of immunohistochemical stains specific to mesothelial cells, such as calretinin and cytokeratin 5/6, allows for the distinction of BCM from other entities [1, 3, 6, 11].

BCM is generally described as a benign entity; however due to the high rate of recurrence (27–75%) some researchers have disputed this point. Recurrence tends to be local and can occur many years later. It has been proposed that BCM acts more as a borderline lesion with potential for transformation to a malignant tumor [1, 5]. Rare cases of malignant transformation have been reported which result in aggressive diffuse malignant mesothelioma [12]. In this respect, some surgeons advocate for aggressive surgery followed by chemotherapy [1, 3, 13]. However, the use of adjuvant chemotherapy and radiotherapy continues to be controversial. The accepted standard of treatment remains complete surgical resection with careful long-term follow-up.
